# Migrants’ Access to Mental Health Services in Italy: The Case of the Transcultural Psychiatric Operational Unit of Catania in Eastern Sicily

**DOI:** 10.1007/s11013-025-09950-3

**Published:** 2025-11-08

**Authors:** Irene Maffi, Simona Carotenuto, Aldo Virgilio

**Affiliations:** 1 University of Lausanne, Lausanne, Switzerland; 2 Psychotherapist and Volunteer Cultural Mediator, Catania, Italy; 3 Azienda Sanitaria Provinciale of Catania, Catania, Italy

**Keywords:** Ethnopsychiatric care, Sicily, Migrants, Recovery, Therapeutic itineraries, Inequalities

## Abstract

This article, based on a collaboration between an ethnopsychiatrist, a psychologist and an anthropologist at the Transcultural Psychiatric Operational Unit (TPOU) in Catania, Sicily, examines how a culturally sensitive approach can support migrants suffering from trauma, depression and other psychological or psychiatric disorders in their recovery and adaptation to the host society. First, we analyse the structure of Italy’s migrant reception system and the specific characteristics of the public healthcare framework in Sicily. Next, we trace the history of the TPOU, detailing patient profiles and the facility’s philosophy of care since its inception. In the second part, through an exploration of five individuals’ therapeutic journeys, we illustrate how access to ethnopsychiatric services has facilitated their recovery and sociocultural integration. Finally, we underscore the disparities in access to treatment opportunities and psychosocial distress prevention programmes in Sicily, highlighting the absence of public facilities capable of providing culturally competent responses to migrants’ social suffering.

## Introduction

In January 2023, there were 5,050,257 foreign residents in Italy (CARITAS, 2023), compared to 4,387,721 in 2013 (CARITAS, 2014). Most of this population lives in the North of Italy, whereas only 200,340 (ISTAT, 2024) of Sicily’s 4,833,329 total residents are officially registered foreigners (ISTAT, 2021). Sicily is mostly a gateway for migrants who come to Europe without a visa from non-European countries. Many cross the Mediterranean on precarious boats, but some of them come from other European countries, where they may have lived for several years. Depending on their country of origin, the journey to Europe can take less than a month or even several years; it can also be the source of severe physical and psychic suffering (Copping et al., 2010; Halluin, 2009; Womersley & Kloetzer, 2018). During the trip, they very often endure several forms of severe hardship, awful violence, torture, economic blackmail and a continuous state of anxiety and fear (Pinelli, 2013; Kobelinsky, 2017, Cordova Morales, 2021). The trip across the Mediterranean can also be a source of traumatic experiences when, for example, migrants see companions die or are themselves victims of a shipwreck and spend hours in the sea before being rescued (Petit and Wang, 2018; Rechtman, 2000; Virgilio, 2020). Because many people start the journey to Europe due to family violence, war, or extensive political violence in their country of origin, their mental state can already be severely compromised before their escape from home.

In this article, we intend to investigate how access to ethnopsychiatric services affects the overall health and social integration of numerous migrants who suffer from psychological distress, traumatic experiences, depression and anxiety. Some^1^ migrants develop psychiatric pathologies in relation to the traumatic experience of their journeys or the difficult conditions of their lives in Sicily. Others who were already suffering from psychiatric disorders before their journeys can arrive in Italy in much worse condition.

Mental disorders and traumatic syndromes among many forced migrants stem from structural violence, inequalities related to class, gender and race, political and armed repression, poverty, neocolonial and neoliberal policies, as well as climate change and natural disasters (Hinton & Good, 2016). The systemic and structural nature of violence and inequality in many contexts highlights the “interpersonal grounds of suffering,” making it fundamentally a “social experience” (Kleinman, Das & Lock, 1997: ix). Individual traumatic experiences and suffering must thus be understood as embedded within a broader intersubjective framework, which is “culturally patterned into recognizably shared forms” within each social group (Kleinman & Kleinman, 1991: 279).

This article examines the case of Sicily, specifically Catania, where a government-run outpatient clinic specializing in transcultural psychiatry has been operational since the early 2000s. Our objective is to demonstrate how a culturally sensitive approach to migrants suffering from trauma, depression, or other psychological and psychiatric issues can aid their recovery and facilitate their adjustment to the host society (Saglio-Yatzimirsky & Wolmark, 2018). We further explore the ethnopsychiatric approach as a form of care rather than merely a cure, as it provides a space where patients can speak in their language and interpret their suffering according to their own cultural terms (Giordano, 2018) and simultaneously create a dialogue with the therapist and her/his professional and social knowledge. Drawing upon emblematic therapeutic journeys of selected individuals, we analyse how access to ethnopsychiatric services has supported their recovery and sociocultural adaptation. In line with other Italian scholars (Beneduce, 2014; Giordano, 2011; 2017), we argue that psychiatrists and psychologists trained to collaborate with cultural mediators (Vargas, 2021) and other professionals can provide the culturally sensitive care migrant patients require. We support the assertion that “the sensibility that has historically characterized Italian psychiatry is not always adequate for working with foreigners, as many clinical models and therapeutic approaches fail to account for the sociocultural transformations experienced by individuals and communities” (Beneduce & Martelli, 2005: 385). Additionally, we acknowledge the considerable challenge posed by widespread cultural, religious and political right-wing ideologies on the therapeutic relationship---affecting particularly Muslim and non-white patients---as they significantly impact Italian psychotherapists working within public mental health services (ibid.).

Although this is true for all health domains (Quaranta & Ricca, 2012), it is even more important for migrants suffering from psychiatric and psychological troubles in that the cultural models that shape their pathological symptoms, interpretation and care can be very different from those of Euro-American biomedicine and psychology. As many authors have theorized (Beneduce, 2014; Coppo, 2000: Kleinman, 1988; Nathan, 1996), without an adequate understanding of cultural differences, health care practitioners (psychiatrists in particular) are unable to offer effective psychiatric or psychological care. However, the Italian public health care system struggles to train culturally sensitive providers and often neglects patients who do not adjust to the local norms and practices, making it impossible or very difficult for them to find treatment (Cardamone and Zorzetto, 2019). The absence of cultural mediators in most health care facilities is but one example of this lack of attention, as well as the absence of systematic training for providers working in government hospitals and outpatient clinics.

In the rest of the article, we will first briefly examine the organization of the Italian reception system for migrant people as well as the public health care system and its specificities in Sicily, for there are important regional differences. We will then retrace the history of the Transcultural Psychiatric Operational Unit (TPOU), where two authors of this article have worked, describing patient profiles and the philosophy of care in this facility since its creation. In the second part, we will examine five patients’ stories in detail, emphasizing how following up with the TPOU changed their lives and facilitated their integration into Italian society.

## Methodology

The three authors met at the TPOU in autumn 2023. Author and Author were already collaborating on a regular basis in this facility as a transcultural psychiatrist and specializing psychologist, respectively. Author is the former head of the TPOU, which is part of the Department of Mental Health under the umbrella of the Provincial Health Authority of Catania. Author is a psychotherapist in training who has been an intern at the TPOU since 2022. She has experience as a volunteer cultural mediator in Campania (in South Italy), where she collaborated with several local associations and performed a year of voluntary civil service at a listening and orientation desk for migrants set up by Caritas in Aversa.

Whereas Author’s and Author’s knowledge relates to their clinical experience with migrant patients coming to the TPOU, Author is an anthropologist who joined them in September 2023, when she began her ethnographic fieldwork in Sicily. Her collaboration with them is part of a larger study focusing on migrants’ access to healthcare services in Sicily. According to the ethnographic method, Author has been attending consultations at the TPOU several days a week during the almost 10 months she has spent with Author, Author and another psychiatrist who works in the same facility. She has fruitfully collaborated with her colleagues, offering her anthropological and linguistic expertise (Arabic, English and French). Author has obtained ethical clearance from the competent health care authorities (Azienda Sanitaria Provinciale) to conduct her research at the TPOU, which is part of a larger facility offering services in other medical specialties, the Outpatient Clinic for Migration Medicine and Health Emergencies (*Ambulatorio per la medicina delle migrazioni e delle emergenze sanitarie*).

For this article, we have selected cases of several patients whom Author and Author have followed for extended periods, as these cases illustrate the critical role of the ethnopsychiatric approach in shaping therapeutic pathways and sociocultural integration in Italy. While the concept of integration has been criticized for reinforcing implicit cultural hierarchies (Quiminal, 1991; Sayad, 1999) and normative state-centred models (Scholten et al., 2022), we define integration as a dynamic and continually negotiated equilibrium involving both migrants and the institutions and civil society of the host country. Within ethnopsychiatry and medical anthropology, the question of integration is complex: it does not merely concern adaptation to the host country’s culture but should also be viewed as a transformative process requiring individuals to rework their cultural roots and past traumas while continuously negotiating between different cultural worlds (Devereux, 1970; Moro, 2010).

Finally, we outline the contributions of each author to the writing of this article. Initial discussions helped define the topic, select relevant material and structure the paper. Author proposed case studies based on his clinical experience and drafted sections on the TPOU and the mental health landscape in Italy and Sicily. Author contributed to analysing five case studies and describing the methodological approach within the clinical setting. Author coordinated the overall writing process, composing significant portions of the introduction and multiple sections throughout the paper. She values collaboration with experienced clinicians specializing in ethnopsychiatric care---especially those outside academia---since their insights, though seldom published, enrich scholarly discourse.

## The Reception System for Migrant People in Italy

Before we discuss the main subject of this article, some description of the reception system for migrants in Italy is necessary.

The reception system in Italy has gone through several changes over the last two decades. Legislative Decree 142/2015, implementing Directive 2013/33 of the European Community, contains regulations about the reception of persons applying for international protection. Its rules have been amended several times, including in 2023 with those introduced by Decree-Laws No. 20/2023 (the so-called Cutro Decree) and 133/2023 regarding the reception of unaccompanied minors.

Foreigners who enter Italian territory irregularly, including after being rescued at sea, are taken to special facilities called crisis points (hotspots): Lampedusa, Trapani, Pozzallo and Taranto. Migrants remain at these facilities for a limited time to receive initial material and medical assistance and undergo identification procedures. Once the first processes at the hotspots are completed, migrants are transferred to government first-reception centres (CPAs) located throughout the national territory. They are designed to offer the first steps in reception and to complete the operations necessary to define the legal position of asylum seekers in Italy (if not completed at the hotspots). If transferring someone to a CPA is not possible, the migrant will be transferred to an extraordinary reception centre (CAS), instituted by local prefectures and entrusted to private entities.

Food, accommodation and minimal services are provided at CPAs and CASs. The Cutro decree reduced the services offered, limiting them to health care, social assistance and linguistic--cultural mediation. The Cutro decree also introduced a third type of extraordinary reception facilities activated by the prefectures, where migrants can be received if there is no room in a CPA or CAS. Here, services are further reduced; in addition to material reception, migrants can only benefit from health care and linguistic--cultural mediation (social assistance is absent).

Unlike the government centres managed by the Ministry of the Interior, the Reception and Integration System (SAI) is coordinated by the Central Service, whose management is assigned to the National Association of Italian Municipalities (Anci) and the Cittalia Foundation. Local authorities activate and implement reception and integration projects (hosting communities and services for their beneficiaries) on a voluntary basis. Reception in the SAI is dedicated to holders of international protection, unaccompanied foreign minors, persons in particularly vulnerable situations, those who have entered Italy through “humanitarian corridors” and Ukrainian and Afghan asylum seekers. Reception is organized in two distinct levels: the first is reserved for asylum seekers, for whom material, legal, health and linguistic assistance is provided; the second is reserved for protection holders and include, in addition to the first-level services, integration services and job orientation.

## The Italian Health Care System and its Regional Specificities

The modalities of access to health protection programmes for foreign citizens in Italian territory are regulated by legal norms that begin with Law 833/78 (1978), which establishes the national health system and the various legislative measures that subsequently defined the terms within which foreign citizens are guaranteed health protection^2^. Although foreign subjects can enjoy most of the rights on a regular basis, the Testo Unico sull’Immigrazione, or *Unique Text on Immigration,* stipulates that “foreign citizens present in the national territory, who are not in compliance with the rules relating to entry and residence, are assured, in public and accredited facilities, urgent or otherwise essential outpatient and hospital care (...) and are extended to preventive medicine programmes (...). The above-mentioned services are provided without charge to applicants if they lack sufficient economic resources, except for the cost-sharing share on an equal basis with Italian citizens”.

## The History of the TPOU

The TPOU is part of the activities of the Mental Health Department of the Provincial Health Authority (*Azienda Sanitaria Provinciale*, hereafter ASP) of Catania and provides out outpatient activities, training and consulting for other ASP departments and private social organizations; it also triggers scientific dissemination of collected clinical data and operational practices.

The TPOU was created in 1998 but began its clinical activities only in 2005. Originally, the staff comprised figures from multiple professions (psychiatrists, psychologists, professional nurses, social workers, sociologists) who committed weekly hours.

After various phases of reorganization, in 2011, the group was consolidated to a multidistrict body formed by the outpatient clinic of Migration Medicine, the TPOU and Penelope Association---a local NGO dedicated to helping women victims of trafficking and male and female irregular workers who are victims of labour exploitation---in a single building in Catania. In this way, multiple professionals from various organizations were able to fill in for those who were no longer available due to retirement or because they were engaged in other assignments. The introduction of the NGO Penelope^3^ made it possible to add professional figures who were absent from ASP’s staff, such as cultural mediators, social workers, educators, legal advisors and experts in identifying victims of sexual and labour exploitation. Since 2011, the TPOU has been operating daily from Monday to Friday, also opening on Tuesday afternoons.

Access to treatment is free for all foreigners who request it, without distinction between regular and irregular immigrants, with the intention of intercepting the psychosocial distress of foreign (mainly non-European) citizens regularly residing in Italian territory, individuals living in state-sponsored communities for migrants and undocumented migrants forced into precarious and uncomfortable living conditions.

The absence of public facilities capable of offering culturally competent responses to the discomfort of migrants in the entire area of Eastern Sicily has led to the outpatient clinic in Catania becoming the only point of reference for a territorial area comprising the provinces of Catania, Enna, Siracusa, Ragusa and Caltanissetta. This highlights the inequality in access to treatment opportunities and psychosocial discomfort prevention programmes.

## The Psychiatric and Social Approach of the TPOU

The construction of the clinical methodology used in the TPOU was meant to make its personnel culturally competent and was developed by reflecting on the following questions: What happens when a migrant patient encounters the national health care system? Are the person’s symptoms and experiences understood adequately? What kind of difficulties do health care practitioners have? Are they able to grasp migrant patients’ personal history and that of their society of origin?

These questions highlight the national health care system’s professional and linguistic shortcomings: providers are generally unable to refer to models capable of interpreting behaviours and psychopathological expressions in light of cultural specificities, and they have difficulties in establishing therapeutic relationships and abandoning ethnocentric judgements because they are not adequately trained to encounter “the other”.

The limitations of Western psychiatry and its diagnostic categories push some practitioners to turn their gaze to methodologies that merge the anthropological (knowledge of cultural specificities) and psychopathological (knowledge of the oscillations between normality and pathology in different cultural contexts) perspectives (Cardamone and Zorzetto, 2022; Nathan, 1996). Within the scientific literature on ethnopsychiatry, numerous practices have been identified that aim to guarantee a treatment that respects the patient's cultural complexity. These include the mediated interview with the presence of a cultural mediator, the clinic of the symbolic translation of the symptom (Nathan, 1986), and the narrative approach that enhances the migratory story as a therapeutic tool. Intervention is often based on a transcultural multidisciplinary team, and on culturally sensitive psychotherapy models, capable of adapting to the patient's cultural beliefs and codes. Other recognized therapeutic instruments include the integration of rituals or religious practices, the use of culturally sensitive diagnostic tools such as the Cultural Formulation Interview (DSM-5), and the recognition of culture-bound syndromes (Hinton and Good, 2016) as central to avoiding ethnocentric readings of distress. Common to all these practices are two key elements: on the one hand, the continuous training of practitioners, and on the other, the valorisation of the patient's explanatory models, i.e. the way in which he/she attributes meaning to his/her suffering. These practices do not represent a fixed protocol, but flexible and situated tools, to be activated on a case-by-case basis. As Beneduce points out: “it is not simply a matter of recognizing the patient’s cultural code, but of enabling work on his subjectivity” (2007: 105). In this perspective, ethnopsychiatry is configured as a clinical and ethical therapeutic approach, whose objective is not assimilation, but rather the subjectification of the migratory experience and the recognition of the patient as the author of his/her own therapeutic narrative.

Clinicians at TPOU chose to follow the ethnopsychiatric approach because they consider it as more appropriate to interpreting non-western idioms of distress (Nichter, 1981) and psychopathological expressions. Ethnopsychiatry offers the instruments to relate these unfamiliar expressions to cultural specificities and create a space where they are legitimate. Specific training has been indispensable to acquire the ability to recognize and deal with the “disorientation” and “loss of references” that clinicians experience when two new and unfamiliar worlds meet (that of the practitioners themselves and those of the patients).

Ethnopsychiatry is based on the general observation that the experience, interpretation and social and therapeutic treatment of mental disorders vary according to cultural contexts; and its privileged object is the “crisis” therapists and patients who come from different cultural worlds experience when they come into contact and confront each other. This encounter forces psychotherapists to question their ethnocentric position to adjust to unprecedented configurations and to introduce into the therapeutic setting multicultural, multilingual, multidisciplinary and multiprofessional actions, incarnated by new figures such as linguistic/cultural mediators, anthropologists, educators, psychologists/psychotherapists and social workers^4^. The participation of these actors allows to produce an unconventional theoretical and practical pluralism of therapeutic strategies.

Letting the “world of the other”, with its culture, religious and therapeutic traditions, food, customs and language, enter the clinical setting and accepting their social belonging creates the space for individuals to be present with their whole “world” and makes it possible to build effective therapeutic itineraries that respect their cultural dignity. Indeed, cultural devices allow individuals to “exist” and “co-exist” and prevent the risk of “losing themselves in the world” (crisis of presence) or “losing the world” (cultural apocalypse) (De Martino, 2019). The clinical approach at TPOU—like at the Centro Fanon (Giordano, 2018)—is not to impose diagnostic categories alien to patients coming from non-European contexts but is to create a dialogue and a space of encounter where “historically and culturally contextualized understanding of trauma survivors can help avoid dehumanization and interpretive violence” (Hinton and Good, 2016: 40).

Ethnopsychiatry makes it possible to overcome the limits of the Euro-American worldview and its inability to welcome the “extraordinary” and “disorienting”, which do not usually find scientific or academic legitimacy. Its privileged object is the crisis experienced by subjects belonging to different mental and cultural contexts once they meet each other. The therapeutic strategy consists of working with the patient to identify and construct the new world implicit in the migration experience and establish the patient’s place in it.

## The Profiles of the TPOU’s Patients

Due to its geographical position in the centre of the Mediterranean Sea, Sicily is the first place of landing for most migrants arriving via the Central African route, which has the coasts of Libya, Egypt and, in the last three years, Tunisia as its points of departure. Migrants land in Sicily either independently or after rescue operations by the Italian Coast Guard or NGOs that patrol the Mediterranean Sea north of the Tunisian and Libyan coasts.

It is well known that those travelling this route via Libya and Tunisia suffer degrading and inhumane physical and psychological violence in almost all cases, which indelibly marks people’s lives and risks leading to psychopathological disorders of varying severity (Medici Senza Frontiere, 2022).

The first specialized facilities offering ethnopsychiatric care in Italy were established in Turin (Centro Fanon) in 1996 and in Milan (Servizio di Etnopsichiatria at Niguarda Hospital) in 2000, followed by the Centro SaMiFo (Salute Migranti Forzati, or Health of Forced Migrants) in Rome in 2006. In Sicily, two non-governmental clinics providing culturally sensitive care exist in Palermo: Centro PENC, founded in 2015, and the Ambulatorio Specialistico per Sopravvissuti alle Violenze Intenzionali (Specialist Outpatient Clinic for Survivors of Intentional Violence), created by Doctors Without Borders in 2021.

The TPOU began its activities in 2005 and has since expanded and adapted its approach in response to the evolving profiles of its patients. Initially, the clinic primarily served permanent residents from Eastern Europe and East Asia. However, since 2011, Sicily’s foreign population has been predominantly composed of individuals from North and sub-Saharan Africa as well as the Horn of Africa.

These persons arrived in Sicily after a long, dangerous journey punctuated by inhumane traumatic experiences. Only practitioners with the professional skills to manage the psychopathological expressions of post-traumatic stress disorder (PTSD)^5^ can face the complexity of the patients’ new experiential dimension, skills which were largely absent among the staff of the government Mental Health Services^6^. The Italian Ministry of Health published guidelines in 2017^7^ regulating assistance and rehabilitation interventions and the treatment of mental disorders for refugees and those with subsidiary protection status who have suffered torture, rape or other serious forms of psychological, physical or sexual violence. However, the Sicilian region has never turned them into local guidelines. This has prevented the regional mental health services from being endowed with the organizational and professional competences necessary to decipher behaviours as well as signs of suffering and possible emotional distress among migrant patients. These manifestations of malaise could be promptly identified and addressed with the appropriate support, shaping the process of migrants’ integration positively, whereas the lack of this support often negatively affects migrants’ lives. Therefore, the intercultural training of health care providers remains a free personal initiative of health care providers. Because most practitioners in government health facilities are overwhelmed by their daily work and often do not know where to receive appropriate training, regional authorities’ lack of interest implies that the large majority are not able to offer appropriate care to migrants^8^.

From 2013 to 2023, 1,228 users (797 men and 429 women) were registered at the TPOU and 10,875 consultations were provided. Most migrants come to the outpatient clinic upon referral, accompanied by social workers of the reception communities for asylum seekers present in the territory, and most are between 16 and 26 years old. The most common pathologies are related to the consequences of the violence and inhumane treatment suffered during the journey and stay in Libya and, lately, Tunisia. The care offered has been functional for the treatment of major depressive disorders, PTSD, psychotic disorders, anxiety management disorders, sleep disorders, behavioural disorders and drug addiction.

Mental Health Services’ difficulty in dealing with the problems described above is related to the fact that providers are unprepared to encounter other cultures and are not able to deal with the complexity of a phenomenon that requires a multidisciplinary and multicultural approach. On one hand, they have to be able to enter into relations with other organizations and psychosocial agencies in the region to coordinate the therapeutic intervention. This can be quite difficult even for ethnopsychiatrists as Giordano has shown in the case of the Centro Fanon in Turin because state institutions delegitimate the approach of the former in the name of a western-based psychiatric model (2018). On the other, highly qualified professionals capable of taking charge of survivors of torture and intentional physical and psychological violence are scarce. Ethnopsychiatrists in particular should be able to penetrate territories of unspeakable suffering and to inhabit survivors’ fragmented and confused worlds; accept and embrace their silences, anger and pain; and support them in their paths towards a new possible world. This is particularly challenging because migrant patients are constantly focused on the past without any prospect of a future.

**Figure 1:**
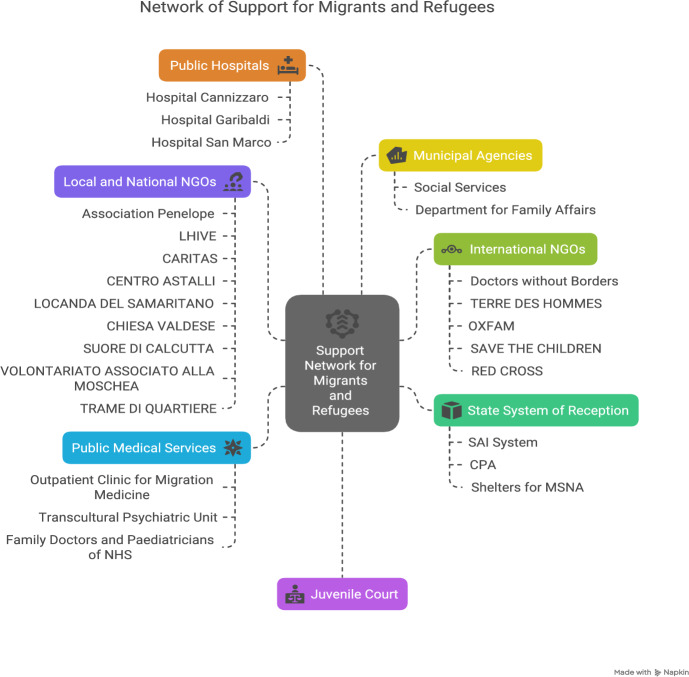
The existing actors offering services to migrants in the area of Catania, among which there are local and international NGOs, religious associations, state-funded communities and government medical institutions (Hospitals and outpatient clinics).

Providing care to migrants necessitates a critical awareness of the therapeutic setting's inherently political nature, as it is a space where individuals engage in “power-laden relationships with authorities, spirits, therapeutic technologies, institutions and nation-states” (Giordano, 2018: 35). Migrants also bear the weight of a “repressed collective past” (Beneduce, 2008: 511), deeply rooted in colonial history and still shaping their psychic and material existence (See Figure 1).

## Clinical Stories of Migrant Patients

In the following section, we will describe five clinical cases we followed at the TPOU that illustrate how the culturally adequate care of migrant patients can be determinant for their health and a process of social inclusion granting their well-being.

Before describing these cases, however, we would like to draw attention to some specificities characterizing follow-up with migrant patients. They often come to the psychiatrist without clearly understanding why they are there and what the psychiatrist will do with him/her. The first step is to openly tell them why they are there and reassure them about the doctor’s role and the confidentiality they will receive. To build a relationship of trust allowing the patient to tell his or her story, it is necessary to take time, provide information clearly and become familiar with the therapeutic setting, where they will meet several therapeutic and social figures, such as the psychiatrist, the psychologist, the linguistic and cultural mediator, the social worker and the anthropologist.

Furthermore, the needs of migrant patients are never singular but inherently multidimensional. They require holistic care that extends beyond medical treatment to encompass mediation with the social environment and various aspects of life in the host country. Their concerns may include housing, employment, residency permits, legal matters, interactions with the judiciary, social workers, national institutions and authorities from their country of origin. At TPOU, clinicians routinely collaborate with local authorities, public medical facilities, migrant reception centres and NGOs to ensure patients find the best possible conditions for recovery. We argue that mental suffering cannot be dissociated from social suffering and the material circumstances in which migrants live. Often, TPOU clinicians extend their work beyond the therapeutic setting to assist patients in securing housing, employment, legal residency or access to medical services. This approach contrasts with Richard Rechtman’s assertion that therapists should not engage in providing social or political support (2006: 178). In Catania, clinicians operate within a network of private and public institutions, leveraging their position and medical authority to coordinate essential services. Given the frequent inability of social services and local authorities to effectively collaborate, the role of TPOU clinicians is particularly crucial in ensuring adequate living conditions for migrants experiencing traumatic disorders.

Ethnopsychiatric care must provide migrant patients with a comprehensive understanding of all factors affecting their lives in the host country. Their physical and psychological health is intimately linked to broader social conditions. Inadequate linguistic and cultural mediation often results in misunderstandings and misinterpretations, preventing access to essential care and services while also obstructing patients’ ability to make informed choices about their lives. Each case examined here illustrates challenges commonly faced by migrants experiencing mental and social suffering in Sicily and, more broadly, in Italy, where ethnopsychiatric services remain scarce and are primarily concentrated in major urban centres in the north (Medici Senza Frontiere, 2022). Key issues include geographical barriers restricting access to services, the absence of culturally competent professionals within medical, legal and administrative institutions, difficulties in interpreting patients’ idioms of distress and social behaviour, as well as challenges faced by migrant children living with foster families.

The five case studies presented here demonstrate the critical role of ethnopsychiatric care in integrating explanatory systems and healing rituals from diverse sociocultural contexts, thereby fostering the recovery of individuals suffering from severe migration-related mental distress.

## Musa^9^

Musa is a young Gambian man who arrived in Italy in 2015, when he was about 17 years old.

He arrived in Sicily after a period of captivity in Libya, where he reports having suffered violence. At first, he lived in Ancona, where he was granted humanitarian protection; then he moved to Gela, a province of Caltanissetta (Sicily), to work as a warehouseman, but it was at that time that he began to present evident psychotic disorders of a dissociative and paranoid type that made it difficult for him to keep his job and good relations with people. For a long time, he looked for a doctor who could help him, but in the Gela area, he found no one to turn to, so a friend told him about TPOU. Here, his demand for care, with its cultural specificities, could be met. Musa lost his father when he was a child. His father was the driver of a car transporting some of his country’s politicians and lost his life in an assassination attempt. When Musa grew up, the same faction that killed his father threatened his cousin, and his mother advised him to leave the country.

During the therapeutic sessions, he complains of being tormented by the people who hurt him in Gambia, but especially in Libya, the people in the latter appearing to him as *presences* who threaten his life by terrorizing him and preventing him working regularly. He has changed jobs several times and reports that every time someone scolds him or shouts at him or has an argument, he is reminded of the violence he suffered in Libya, which he relives through flashbacks and intrusive thoughts.

In TPOU, he was supported with pharmacological treatment to reduce the symptoms of the psychotic disorder but above all he received a culturally appropriate listening. To travel to the outpatient clinic in Catania for periodic clinical interviews, Musa faced long bus journeys without ever missing an appointment. The psychiatrist slowly reconstructed his story and helped him regain confidence in his ability to integrate socially and work in Italy. After a difficult economic period in Gela, he moved to the Locanda del Samaritano, a structure located in Catania and managed by religious volunteers, which took charge of social planning and his job placement at a hotel in the city. Musa works there as a dishwasher, a job that has allowed him total economic autonomy and the possibility of living in his own apartment. Today, his conditions have improved considerably: he expresses plans for the future, such as obtaining a driver’s licence to become a truck driver and helping a younger brother, also in Italy, who is currently living in serious hardship.

Musa's case underscores how access to TPOU was a pivotal moment: his suffering was acknowledged within his biographical and cultural framework, rather than being reduced to a mere psychiatric disorder. The ethnopsychiatric team addressed his condition through both pharmacological and symbolic interventions, emphasizing his personal narrative and reconstructing a shared frame of meaning. His therapeutic journey also illustrates the inseparable link between social planning and clinical care; his placement at Locanda del Samaritano and subsequent employment in Catania exemplify a holistic model of patient support. Through this approach, Musa regained his sense of agency and capacity for future planning, demonstrating how ethnopsychiatric practices facilitate not only symptom stabilization but also the reconstruction of identity damaged by migratory trauma.

An important aspect of Musa’s journey was the recognition of the need to move to another city and to reconsider his job prospects. This move marked his active engagement in therapeutic change, highlighting the transformative dimension of the ethnopsychiatric device.

## Blessing

Blessing is a Ghanaian woman who arrived in Italy in 2021 and was taken in with her two- and ten-year-old children in a SAI community for families in Vittoria (close to Ragusa).

She landed in Sicily from the Libyan coast. Her partner, the two-year old child’s father, was supposed to join her but was rejected several times by the Libyan police and decided to return to Ghana.

Social workers in the community that received her requested a consultation because a few months earlier, she had subjected her two-year-old child to clandestine circumcision. This circumcision, carried out without respecting any sanitary norms, generated an infection of the genitals that seriously endangered the child’s life and physical integrity. The case was reported to the social services and the Juvenile Court of Catania, which opened a file to protect the child. The mother was allowed to remain in the community with her son because she was in a “protected place”. Blessing reported that she regretted having given in to the pressure from the child’s father and family of origin to contact members of the Ghanaian community in Sicily to perform the circumcision in the traditional manner. She said she was very worried about the legal consequences of having transgressed Italian laws, and at the same time, she was afraid that members of the Ghanaian community might act threateningly towards her.

During the interviews, a strong distrust of her compatriots emerged, even in their role as cultural language mediators, so much so that she preferred mediation in English by Italian operators, but she also distrusted the Italian institutional figures for fear that they might decide to take her children away from her.

At the TPOU, Blessing slowly found the strength to trust and talk about how the pressure from her partner and family members in Ghana pushed her to practice a clandestine circumcision rite, making her feel inadequate as a mother and disrespectful of her religious norms. Clinicians, being able to reconstruct the woman’s intentions and motivations, became a point of reference and mediation between Blessing and the institutions, helping shed light on the cultural component that, because Blessing had not found acceptance in the Italian social context, pushed her to rely on an illegal action to fulfil the moral duties of a good mother in her home context. This allowed Blessing to demonstrate her adequacy as a mother and to relocate herself in the social discourse with greater awareness of what her home culture requires of her and what is necessary to build an adequate social and working life for raising her children in Italy.

A decisive step for Blessing was the gradual abandonment of the conviction that she could not trust anyone. The realization that belonging to the community of origin was no longer sufficient in the new context made it possible for her to trust people outside her family and ethnic group, paving the way for a process of maternal rehabilitation and self-protection in the host society, as well as for the protection of her son.

Blessing's case highlights the role of ethnopsychiatry in dealing with conflicts between cultural norms of the country of origin and the laws of the host country. The act of clandestine circumcision is understood not only on a legal level, but also as an outcome of family and cultural pressures. At the TPOU, Blessing found a non-judgmental space that allowed her to process the meaning of her gesture, reconstruct her maternal position and establish a dialogue with the institutions. The team acted as a mediator between cultural codes, fostering a greater awareness of her parental role in Italy.

## Maryam

Maryam is a teenage girl who attends high school with excellent results and who manifested severe social withdrawal, inappetence, depressive symptoms and an obvious stutter.

She was born in Kinshasa, Congo, to parents who were, according to her, affectionate and well-educated. For unspecified reasons, when she was nine, her parents entrusted her to a self-styled aunt. After crossing the desert, she arrived in Libya and then in Sicily in 2016. She was entrusted to an Italian family, with whom she lived for a year in Tuscany and then in a town in eastern Sicily. The family climate is described as affectionate, and she is so close to them that she regrets not having childhood memories with them like her peers with their parents.

She came to the outpatient clinic because she was referred by the psychiatrist of the Syracuse child neuropsychiatry service, who had been following her since 2017. The doctor, due to the child’s increasingly avoidant attitude even towards therapy, thought it appropriate to refer Maryam to TPOU for an ethnopsychiatric consultation.

Maryam prefers to express herself in French at psychotherapy sessions, partly because, as she later clarified, she is afraid of forgetting this language and losing contact with her culture of origin. Describing her school life and her relationships with peers and teachers, she reports that she used to hang out with her classmates but has recently become withdrawn, preferring to spend the days alone in her room and, once she has finished her studies, to listen to music and watch videos from her country. She expresses a desire to get in touch with her biological parents and siblings on a daily basis but is also afraid of this rapprochement’s possible consequences. She is very homesick for her country of origin but realizes the opportunities she has here and feels that she belongs to both cultures.

Maryam admits that she is worried about school, especially not being able to socialise with peers. She wonders about her future university studies and her job possibilities; she would like to work for the UN to help women and children in need. She faces difficulty in reconciling the two realities in which she lives and her two cultures.

After several attempts, she managed to get in touch with her sister, who lives in Tunisia and, thanks to the Congo embassy, her biological parents, whom she started to hear from regularly. She shows ambivalent feelings towards them and asks why they forced her to leave.

But now that she has resumed relations with them, she feels reproached for her behaviour, which her parents consider too “Westernized” and not respectful of her country’s religious traditions.

As the talks in the TPOU continue, Maryam agrees to speak in Italian and not to use language mediation. Her mood has improved, and some symptoms, such as social withdrawal and stuttering, have disappeared.

The psychotherapy sessions led her to address the cultural dimension and the relationship between her Congolese and Italian cultures, enabling her to search for her own cultural identity.

Mariam’s therapeutic itinerary allowed her to reflect on her identity and to separate herself from family pressures without cutting off the bond with her family of origin. The processing of this transition allowed her to recognise in the adoptive family not a substitute, but a new possibility of support and appreciation, thus opening the way to a satisfying life project in Europe.

Maryam's case highlights the effectiveness of ethnopsychiatry in supporting young migrants facing identity crises. Her early separation from family, the trauma of migration and her ambivalence between cultural belonging and integration into Italian society led to depressive symptoms, social withdrawal and stuttering. At TPOU, her ability to initially communicate in French preserved her cultural connection, helping her feel accepted in her complexity. Over time, clinical work enabled her to symbolically reframe her migration trauma and identity struggles, transitioning from a fragmented self-perception to a more integrated one. Ultimately, Maryam emerged as an active agent in shaping her future, maintaining a critical yet affective bond with her heritage.

## Joyce

Joyce is from Nigeria and is about 30 years old. She has three children in her home country and two in Italy, M. (born in 2018) and J. (born in 2021).

In June 2021, J.’s father abandoned them to look for work in another city, and Joyce found herself alone with the children in a desperate economic situation for many months. In February 2022, she went into a club in Catania to ask for help from policemen who were there, trying to explain that she had nowhere to go and had no food. They made a report to the Police Headquarters, and Joyce and her children were taken to a shelter. The children appeared very neglected and undernourished to the shelter workers. In particular, J. presented dermatitis all over his body and candida on his mouth. On the first day, Joyce manifested strong emotional dysregulation, which resulted in behaviour dangerous for the child the next day. She did not want to get out of bed; cradled him with repetitive movements that worried the educators, holding him tightly to her and talking to herself; and became agitated if the educator tried to take the baby out of her arms, holding him even tighter and risking suffocating him.

The social workers then decided to call the 118 emergency services to take Joyce to the psychiatric department of the Cannizzaro hospital in Catania for compulsory medical treatment (*Trattamento Sanitario Obbligatorio*).

The reporting community also gathered more information about the woman, discovering that she had arrived in Italy three years earlier and was placed in a protected community near Matera, where her daughter, Mariam, was born, as a trafficking victim. She was assisted until she moved with her partner to Syracuse, but when he left for Malta to look for work, she started a relationship with J’s presumed father and moved to Catania.

The case was immediately reported to the Juvenile Court of Catania, which, considering this information, immediately suspended Joyce’s parental responsibility and placed the children in the care of two different families, declaring the children’s adoptability procedure open because the woman’s conditions of personal and social distress were incompatible with her suitability to care for the children at a very young age.

After the TSO discharged her a month later with a diagnosis of unspecified psychotic disorder, Joyce was hosted by a community for victims of human trafficking in a village near Catania, where, with the TPOU’s mediation, she began a path to regain custody of her children.

She demonstrated good adaptation to community life and good psychopathological compensation. She expressed great suffering from the separation from her children but always in an adequate and contained manner. This made it possible to start a series of meetings with the children in a protected environment with the Social Services of Catania.

During the meetings, Joyce showed appropriate behaviour with respect for her children and was attentive to their emotional states and respectful of their time.

Thanks to the protected meetings and the observation by TPOU’s workers, it was possible to rebuild a healthy and appropriate relationship between Joyce and her children, which had been put in crisis by the removal. Joyce’s children were permitted to return to live with their mother, albeit supported by the host community workers.

Joyce now lives with her children in the community and is able to reconcile work and childcare.

For Joyce, the pain of being separated from her children, who were entrusted to others and who rejected her, was the pivotal point of her change. The therapeutic framework allowed her to transform the waiting period into a time of personal processing, fostering the emergence of new relational modalities that were useful in rebuilding the bond with her children, with a view to regaining custody and the possibility of receiving their affection again.

Joyce's case illustrates the impact of an ethnopsychiatric approach on migrants facing psychological and social vulnerability. Her psychic distress stemmed from a history of separation, abandonment, marginalization and family disintegration. Her disorganized behaviour towards her youngest son cannot be understood outside the context of migration trauma and the collapse of primary support structures. Ethnopsychiatry provided Joyce with a clinical relational space where she could process her cultural and emotional rupture, facilitated by mediation between cultural codes and collaboration among clinicians, reception centres and social institutions. Reuniting with her children, supported by integrated clinical and social efforts, was not simply a therapeutic goal but a means of reconstructing a psychic and social framework that restored her parental desire and responsibilities.

## Samira

Samira is a woman who was born in Ghana in 1993 and arrived in Italy via Libya in 2015. She had no education; lived with her father, stepmother and four brothers; and worked in the fields. Hers is a very tormented story of abuse in the family and then in Libya. When Samira was very young, her stepmother decided to give her away in marriage for money to a man she did not want. Samira tried to escape him, but he raped her on her way to a river.

According to local cultural interpretations, that river is inhabited by female spirits, water jinns, who, as a result of that rape, cursed her, causing her somatic symptoms and various troubles. For example, she thinks that she cannot have children because of this curse.

Following this episode, Samira left for Italy via Libya, where she was exploited in a ghetto and then made to embark for Sicily.

The reasons for her coming to TPOU were dissociative amnesia, depersonalisation, somatic symptoms, motor co-ordination and speech articulation disorders, tremor and dystonia. Since the day of her rape, Samira lost control of her neck, which escapes her “like a child to her mother,” as she says in a session.

Samira began a motor rehabilitation programme that has lasted for many years and yielded some improvements, but the desire to return to Africa was ever stronger in her. She thought that the only way to heal was to perform a ritual involving the killing of a ram in her name that would purify and rid her of the curse afflicting her. As explained above, therapeutic work in ethnopsychiatry and ethnopsychoanalysis takes place through several reference figures that constitute for the subject a real reference group, as in a village. The linguistic mediator, the psychologist, the anthropologist and the educator discuss with the patient, question her and allow her to question them on her experience and specific ideas about the events to stimulate a process of reorientation of the subject in the context that surrounds her.

This was the case with Samira, who did not speak Italian or other European languages and was always supported by cultural mediators from her country or people who spoke her mother tongue as well as the educators in her community. One of the mediators coming from the same country formed a very strong bond with her and, seeing her desire to return to Ghana to perform the healing ritual she wanted so much, helped her by accompanying and hosting her, along with his family, in their country of origin. He wished to protect her from the malicious intentions of her stepmother, who was still ready to harm her and take advantage of the little money Samira received for her civil invalidity.

Once back in Italy, Samira was better off, but she found the conditions of little independence, which she experienced in the community, constrictive, and she continually complained that she was not free to choose whom to be with or whom to go out with; she also had a strong desire for motherhood, which was prevented by the discovery that she had HIV.

In the community, workers tried to make her aware of the possibility she would have to find a suitable job and perhaps become independent. Her story and condition had led to her being granted international protection with a five-year residence permit, and returning to Ghana would have been an unnecessary risk for her given her difficulty in finding the necessary medicines to treat herself. However, as time went by, Samira became less satisfied with her choice to stay in Italy, so during the therapeutic sessions, she was able to outline a plan to return to her country, taking into account her resources and fragilities.

In Ghana, she has an uncle who, although an invalid, loves her and could protect her socially and her mother, who had been removed from her father’s household.

With the money she received in Italy for economic support, she decided to buy land and build a house with a vegetable garden to cultivate and sell the products of her land. This was also possible thanks to the contacts with local agencies that the cultural mediator who helped her so much had. Samira’s return to Ghana thus become possible after a long and difficult migratory journey, with the protection and support of those who, with human respect and professionalism, helped her on a path of personal redemption and greater awareness.

In her therapeutic journey, Samira was able to realise the limit of her autonomy and full social integration in Italy. The transformation of this awareness into a desire to return to Ghana represented a fundamental step, thanks to which the patient was able to regain an active position, recovering her autonomy and escaping the passivity of choices imposed by others.

Samira’s case demonstrates how ethnopsychiatry validates and integrates cultural beliefs surrounding trauma, supporting a care process that acknowledges bodily, symbolic and relational dimensions. Her physical and dissociative symptoms were not only addressed clinically but were also situated within a culturally shared framework---including her belief that she was the victim of a curse. Through collective work involving cultural mediators, educators and therapists, Samira found a space to process her story and make informed choices. The decision to return to Ghana for a traditional ritual was embraced as part of her therapeutic and identity reconstruction journey. Ethnopsychiatry empowered her to reconnect with her roots, transform her suffering into a resource and build an autonomous life trajectory, in alignment with her cultural values and aspirations.

## Conclusion

Based on the five clinical narratives analysed, we assert that access to culturally competent mental health services is fundamental to facilitating migrant patients’ recovery and enabling them to lead dignified lives in their host country. The ethnopsychiatric approach---which includes multiple professional figures---elicits a more competent interpretation of migrants’ mental disorders and a therapeutic itinerary in which suffering individuals engage in a more symmetric exchange with therapists. In the ethnopsychiatric setting, representations, experiences and practices of foreign patients intersect with those of therapists, cultural mediators and anthropologists. When this encounter occurs, patients typically adhere to the therapy and rework their past lives, which they are often trapped in, to reimagine their future.

Ethnopsychiatry restores an active role to the patient: no longer a spectator but a co-constructor, together with various actors, of her own path to care and well-being, reaffirming the right to health as a fundamental right.

The ethnopsychiatric experience at the TPOU also emphasizes the necessity of collaborating with government institutions and NGOs to ensure that patients have living conditions in which recovery and sociocultural adjustment to the host society are possible.

Musa’s case underscores the severe limitations in accessing adequate mental health services in Eastern Sicily and beyond, exacerbated by migrants’ frequent inability to regularly travel to distant facilities for treatment. Many lack the financial resources to cover transportation costs, public transport systems are insufficient, and when employed, migrants often work long hours for low wages with no paid leave. The absence of government-funded, culturally sensitive mental health services equipped with cultural mediators—except in Catania—creates significant disparities in treatment and reinforces inequalities across different areas where migrants reside.

Blessing’s case highlights the crucial mediating role that TPOU plays between migrant patients and local institutions. This underscores the need for healthcare professionals trained in migrant care and aware of the ways cultural specificities shape the interpretation of clinical and social contexts. In Blessing’s case, it was only through open and attentive listening that a tragic misunderstanding regarding her parenting abilities and mental health was resolved.

Maryam’s case illustrates the profound difficulties a teenage migrant girl may face in reconciling her origins with the culture to which she belongs, as well as the importance of attentive listening in guiding her towards greater self-awareness and acceptance of her dual cultural identity. This approach enables her to make informed choices---determining what aspects of her heritage to retain and what to relinquish---so she can construct her own pathway towards integration into the society in which she lives and wishes to remain.

Joyce’s case tells the story of a woman pushed to the limits of her physical and mental resilience, unable to express herself adequately, disoriented in an unfamiliar cultural setting and struggling to understand its norms. Seeking help from the police---whom she perceives as local authorities---her request is misinterpreted, and she is wrongly accused of failing as a mother. Without knowledge of the Italian language, her gestures are misunderstood, and in the panic of potentially losing her children, she clings to them desperately---leading to a complete breakdown. At the TPOU, however, she finds a space free of prejudice, where she is heard and supported with compassion and trust.

Finally, Samira’s case demonstrates how migration can sometimes end in disillusionment, leading individuals to reconsider their decision and ultimately return to their country of origin with a deeper awareness of the challenges of adapting to a new cultural environment. Samira’s experience represents one example of return migration, but many other migrants share similar sentiments, longing to return home due to nostalgia and the lack of sufficient guarantees of well-being in Europe.

Drawing on our experience at TPOU in Catania, we argue that, for the many migrants who have endured extreme violence in their country of origin and/or during their journey to Europe, access to culturally sensitive public mental health services is essential to their well-being and integration. We also contend that expertise in ethnopsychiatry, along with a broader culturally sensitive approach to healthcare, is fundamental to delivering appropriate services. However, disparities in access to mental health support remain widespread across Sicily, limiting migrants’ ability to receive necessary care. These inequalities pose serious risks to many individuals’ migration trajectories and their overall stability. Finally, we would like to emphasise that this article contributes to the knowledge of mental health services for migrants in Sicily, a region that is largely understudied, despite being a crossroads for migratory flows in the central Mediterranean.

## Footnotes

1 In 2017, the Italian Ministry of Health issued official guidelines for refugees who suffer from various psychic disorders where it is clearly stated that « Applicants and holders of international and humanitarian protection, henceforth RTPs, are a population at high risk of developing psychopathological syndromes due to the frequent incidence of stressful or properly traumatic experiences. They are people forced to leave their countries generally to escape persecution or the real risk of suffering persecution. They may also flee contexts of generalized violence brought about by wars or civil conflicts in their home countries. Moreover, during the migration journey, they are often exposed to additional dangers and traumas brought about by the dangerous nature of these journeys, which can take the form of situations of exploitation, violence and aggression of various kinds including sexual violence, malnutrition, inability to be treated, psychophysical humiliation, detention and refoulement » (2017 : 6) They are unfortunately only rarely applied by Italian regions (Medici Senza Frontiere, 2022).

2 The laws defining foreign subjects’ right to health are the following: Laws 33/80 (1980), 489/95 (1995), 40/88 (also known as the Law Turco-Napolitano), 189/2002 (also known as the Law Bossi-Fini) and 286/98 (1998) or Testo Unico Sull’Immigrazione (Unique Text on Immigration), which has been amended several times over the last few decades.

3 The Penelope Association stopped collaborating with the unit at the beginning of the pandemic (2020).

4 The model was the therapeutic setting of the Centre George Devereux, founded in Paris in 1993 by Tobie Nathan (2024).

5 We are aware of the critical debate about PTSD (Hinton and Good 2016), but we consider it an elastic category, a kind of Weberian ideal type, allowing us to classify several psychic disorders related to the experience of extreme and prolonged forms of violence.

6 We base this statement on the long professional experience and knowledge of the field of Autor as well as the interviews Author made with numerous doctors, psychologists and social workers she met during her research in Sicily. See also : ISMU 2025.

7 These guidelines are available online: https://www.salute.gov.it/imgs/C_17_pubblicazioni_2599_allegato.pdf.

8 This assertion is based on Author’s knowledge as he has been working as psychiatrist in Sicily for around thirty years and acted and still acts as trainer for many younger colleagues as well as for officers of various Sicilian municipalities and social workers employed in the government reception system. See also : UNICEF 2022.

9 All names are pseudonymes.
